# Neurological Complications in Pregnancy and the Puerperium: Methodology for a Clinical Diagnosis

**DOI:** 10.3390/jcm12082994

**Published:** 2023-04-20

**Authors:** Merlino Lucia, Matys Viviana, Crognale Alba, D’Ovidio Giulia, Della Rocca Carlo, Porpora Maria Grazia, Titi Luca, Viscardi Maria Federica, Volpicelli Agnese Immacolata, Piccioni Maria Grazia

**Affiliations:** 1 Department of Maternal, Infantile and Urological Sciences, University of Rome La Sapienza, Viale del Policlinico 155, 00161 Rome, Italy; viviana.matys@uniroma1.it (M.V.); alba.crognale@uniroma1.it (C.A.); g.dovidio@uniroma1.it (D.G.); mariagrazia.porpora@uniroma1.it (P.M.G.); mariafederica.viscardi@uniroma1.it (V.M.F.); volpicelli.1800695@studenti.uniroma1.it (V.A.I.); mariagrazia.piccioni@uniroma1.it (P.M.G.); 2 Department of Medico-Surgical Sciences and Biotechnologies, Sapienza University, 04100 Latina, Italy; carlo.dellarocca@uniroma1.it; 3 Department of Anesthesia and Intensive Care Medicine, Sapienza University of Rome, 00161 Rome, Italy; luca.titi@uniroma1.it

**Keywords:** obstetrics, neurology complication, emergency

## Abstract

Neurological complications in pregnancy and the puerperium deserve particular attention from specialists due to the worsening of the clinical picture for both the mother and the fetus. This narrative review of existing data in the literature aims to analyze the most common “red flag symptoms” attributable to neurological complications such as pre-eclampsia (PE), eclampsia, HELLP syndrome, posterior reversible encephalopathy syndrome (PRES), cerebral vasoconstriction syndrome (RCVS), stroke, CVS thrombosis, pituitary apoplexy, amniotic fluid embolism and cerebral aneurysm rupture, with the aim of providing a rapid diagnostic algorithm useful for the early diagnosis and treatment of these complications. The data were derived through the use of PubMed. The results and conclusions of our review are that neurological complications of a vascular nature in pregnancy and the puerperium are conditions that are often difficult to diagnose and manage clinically. For the obstetrics specialist who is faced with these situations, it is always important to have a guide in mind in order to be able to unravel the difficulties of clinical reasoning and promptly arrive at a diagnostic hypothesis.

## 1. Introduction

Women in pregnancy and puerperium with acute neurological symptoms represent a particularly critical category of patients whose diagnostic and therapeutic management must also consider the health of the newborn. The pathogenetic mechanism of these conditions results from the hormonal balance that is different in non-pregnant, pregnant and postpartum women. High estrogen levels stimulate the production of coagulation factors, increasing thromboembolic risk [1]. Normal pregnancy is characterized by higher concentrations of factors VII, VIII and X, the von Willebrand factor and fibrinogen, as well as lower concentrations of free protein S [2]. At the same time, increased plasma and blood volume are conditioning factors for the increase in pressure values and the development of hypertension [3]. Increased progesterone concentration in late pregnancy tends to increase venous wall distention and the risk of small-vessel arterial bleeding [4]. In the postpartum period, on the other hand, there is a drop in high estrogen levels. These alterations can result in changes in brain bioelectric activity and/or cerebral circulation, causing seizures, transient or permanent cerebral ischemia, intraparenchymal or subarachnoid cerebral hemorrhage and cerebral venous thrombosis. The most common neurologic complications of a vascular nature in pregnancy are due to:

- *Cerebral venous sinus thrombosis (CVST)*, which is an uncommon cause of stroke in the general population. Females are more affected than males, and this gender-related pathogenesis is related to pregnancy and the postpartum [5].

- *Reversible cerebral vasoconstriction syndrome (RCVS)*, which is due to multifocal arterial vasoconstriction and dilation. It is most commonly reported 1–3 weeks up to 6 weeks after a normal, uncomplicated pregnancy [6], and female reproductive hormones are considered to play an essential role in its pathogenesis [7].

- *Posterior reversible encephalopathy syndrome (PRES),* which is a neurotoxic state in which subcortical vasogenic edema occurs within the brain resulting in neurologic sequelae [8]. It has been associated with seizures, encephalopathy, headache and visual impairment, and generally occurs in settings of acute renal failure, hypertension, cytotoxic medications, autoimmune diseases or pregnancy-related disorders (pre-eclampsia or eclampsia) [9].

- *Eclampsia*, defined as the occurrence of one or more generalized tonic–clonic convulsions unrelated to other medical conditions in women with hypertensive disorders of pregnancy [10].

- *HELLP (Hemolysis, Elevated Liver enzymes, Low Platelet count) syndrome,* which is the extended spectrum of severe pre-eclampsia and is associated with high mortality. A large proportion of mortality can be attributed to catastrophic central nervous system events. [11].

- *Rare complications,* such as cerebral cavernous malformations (CCMs), stroke, amniotic fluid embolism, pituitary apoplexy, etc.

Currently, neurological complications in pregnancy and puerperium overlap greatly with one another, and there is often confusion in distinguishing them as there are many common points. Confusion, however, wastes valuable time, which is the main factor influencing the outcome. The aim of our review is to give the obstetrician during their shift in the delivery room practical guidance to follow when faced with neurological complications in pregnant women and in puerperium, based on quality scientific evidence. Our purpose is to help the clinicians to quickly recognize or at least suspect such conditions and to guide them both in the diagnostic and therapeutic phases.

## 2. Discussion

The diagnosis of a neurological complication of a vascular nature in pregnancy should be confirmed by clinical and paraclinical manifestations, the associated risk factors and the neurological examination. Imaging investigations are extremely important for confirming the diagnosis and establishing the correct management and treatment. Below, we summarize the main diagnostic neurological conditions involved in pregnancy and puerperium.

### 2.1. Cerebral Venous Sinus Thrombosis (CVST)

CVST represents an important cause of stroke to be considered, especially in women during pregnancy or postpartum. It occurs in 2–57% of pregnancy-related strokes, with the majority of episodes taking place during the postpartum period [12]. The mortality rate is 11.63% [13].

Risk factors include cesarean section, older maternal age, concurrent infection, dehydration, traumatic delivery, anemia, elevated homocysteine concentration and low cerebrospinal fluid (CSF) pressure caused by dural puncture for neuraxial anesthesia [14]. Most patients present with a diffuse, increasing and continuous headache, although 10% present with a severe headache of the stabbing type. Other symptoms include dizziness, nausea, seizures, papilledema, signs of laterality, lethargy and coma [15]. The clinical picture may vary, taking into account the position and size of the dural sinuses and draining veins involved, the presence of collateral circles, the effects of intracranial pressure and any associated cerebral venous hemorrhages and infarcts. Symptomatology may also change over time. With regard to instrumental diagnostics, computed tomography (CT) without contrast medium is often negative, but in 30% of cases it may show indirect signs of infarction or thrombosis. Venous infarcts often undergo hemorrhagic transformations. The diagnostic investigations of first choice to diagnose CVST are cerebral magnetic resonance imaging (MRI), magnetic resonance venography (MRV) or CT venography (CTV) with spin-echo sequences. Contrast medium may be helpful for the MRV since non-contrast MRV can have false positive findings, even if gadolinium contrast may only be given after delivery since no clear evidence of its safety during pregnancy is currently available. [16] A cohort study performed in China shows that the most common sites involved in CVST cases are the superior sagittal sinus and the transverse sinus [13].

It is important that all patients suspected of having CVST undergo screening for prothrombotic states, a study of the mutations of prothrombin and an evaluation of the levels of antithrombin III, S and C proteins, homocysteine and antiphospholipid antibodies [17]. These laboratory data are beneficial and essential for women with other associated risk factors or a history of thromboembolism.

Treatment with unfractionated or low-molecular-weight heparin is recommended in the acute phase [18]. Enoxaparin, a low-molecular-weight heparin, provides a good safety profile and does not cross the placenta or leave the body through breast milk [19]. The newer European guidelines recommend antiepileptic therapy in patients with supratentorial lesions and seizures at presentation [20]. Those requiring longer-term antiepileptic therapy require consideration about both their antithrombotic regimen and potential for childbearing. Acute treatment outside of the context of severe thrombophilia is followed by 3 to 12 months of anticoagulation with a VKA or low-molecular-weight heparin. The newer European guidelines recommend avoiding non-vitamin K antagonist oral anticoagulants (NOACs), particularly during the acute phase. There is no mention of the role for ongoing thromboprophylaxis with antiplatelet agents, though both the American Heart Association/American Stroke Association and the updated European guidelines give a weak recommendation for low-molecular-weight heparin prophylaxis in the context of future pregnancies [21].

A painkiller can be added [22], as well as anti-edematous or antihypertensive treatments.

### 2.2. Reversible Cerebral Vasoconstriction Syndrome (RCVS)

RCVS is a rare clinical entity, and its incidence is unknown. Approximately 0.26% of patients presenting to an emergency headache clinic have been diagnosed with RCVS [23]. It is more common in women approximately 42 years old (10–76 years) [24], and some studies show that 7–9% of the patients had RCVS in the postpartum period (within one month from delivery) [23]. RCVS is associated with a range of conditions, not only postpartum but also with the use of immunosuppressive drugs and vasoactive substances (such as phenylpropanolamine, serotonin reuptake inhibitors or cocaine), as well as endogenous vasoactive substances, catecholamine-secreting tumors, cranio-cervical arterial dissections and other conditions [25].

It is characterized by the sudden onset (reaching peak intensity within <1 min) of an acute, severe and explosive headache of the thunderclap type [26] and reversible cerebral vasoconstriction. The headache often has a posterior onset and can be accompanied by nausea, vomiting, a confusional state, photophobia, phonophobia and visual changes [27]. Generalized tonic–clonic seizures and focal deficits have been reported as well [28]. The daily recurrence of episodes of sudden-onset, high-intensity headache with a duration of several weeks after the first episode is almost pathognomonic [27].

RCVS is diagnosed if a patient fulfills the following criteria:

(a) Segmental cerebral artery vasoconstriction seen on the Magnetic Resonance Angiography (MRA);

(b) No evidence of subarachnoid hemorrhage;

(c) Normal CSF analysis (protein, leukocytes, glucose);

(d) Severe headache with/without neurological findings;

(e) Reversibility of angiographic abnormalities within 12 weeks [29].

MRI and MRA of the brain are investigations that allow us to understand the cause of a severe headache; in fact, they reveal the typical characteristics of RCVS pictures. However, it must be emphasized that the gold standard for diagnosing RCVS is digital subtraction angiography (DSA). It is with this type of examination that we can observe the multifocal segmental vasoconstriction of large and medium-sized brain vessels; the problem with this technique is that it is an invasive examination [30]. The cephalea in RCVS can be mistaken for a sentinel headache from an aneurysmal subarachnoid hemorrhage. When seizures or focal neurologic deficits develop, these almost always follow the headache. Symptoms usually regress in 2 to 3 months. Although RCVS has a favorable outcome in most cases, there are several reasons that can cause a fatal outcome in postnatal patients. These include subarachnoid and intraparenchymal hemorrhage and cerebral ischemia.

In a case series, convexity SAH was present in 33 percent of patients with RCVS [28] A small percentage of patients with RCVS also have cervicocranial arterial dissections. In patients without an infarction, the disease resolves spontaneously. With regard to neuroradiological investigations, it is important to recognize that RCVS is a dynamic process. CT scans and direct or indirect angiographic evaluations (CT angiography and MRI) are useful, but in some cases, they may be normal during the course of the disease. Noninvasive investigations, such as CT or MRI with angiographic sequences, are positive in about 80% of patients and can sometimes show areas of alternating arterial dilatation and vascular constriction that may be indistinguishable from those seen in vasculitis. In these cases, vessel wall MRI can be useful to distinguish the two conditions [31]. Transcranial Doppler can be used to monitor the resolution of vasoconstriction and the normalization of flow velocities. Edema can often be seen in the anterior hemispheres/frontal lobes too, and can be rarely associated with foci of acute hemorrhage.

There is no proven or established therapy for RCVS [25,32]. The treatment involves identification and removal of precipitants, symptomatic management with bed rest, analgesia, consideration of calcium-channel antagonists and avoidance of glucocorticoids [33]. The pain of RCVS-associated headache is extreme, so the use of painkillers is recommended, but triptans and ergot derivatives are contraindicated because of their vasoconstrictive actions [34]. Patients with clinical progression should be admitted to intensive care and have careful neurologic and blood pressure monitoring undertaken [35]. Interventional neuroradiological techniques should be reserved for severely deteriorating cases [33].

### 2.3. Posterior Reversible Encephalopathy Syndrome (PRES)

It is a syndrome characterized by headache, seizures, encephalopathy and visual disorders due to reversible vasogenic edema detectable by neuroradiological (CT or MRI) investigation of the brain, first described by Hinchey in 1996 [36].

The suspicion of PRES arises in patients with acute arterial hypertension, pre-eclampsia or eclampsia, renal disease, sepsis and in subjects treated with immunosuppressants and other drugs [36]. Symptoms develop rapidly and have no prodromal period beforehand, progressing rapidly within 12–48 h. Approximately 90% of patients present with epileptic seizures that tend to have a focal onset and secondary generalization with tonic–clonic movements. Usually, the seizures are preceded by vision changes with bilateral visual blurring or non-distorting headache [37]. Vasogenic edema is mainly located in the occipital lobe, and in fact, about 40% of patients present visual symptoms such as visual hallucinations, blurred vision, scotomas and diplopia. On the other hand, 1–15% of patients have transient cortical blindness, where the retinal and pupillary components do not show anomalies. Most patients are confused and have memory deficits.

A CT scan will show edema in only 50–60% of patients, so a brain MRI should be performed when PRES is suspected. Encephalic MRI reveals focal edema, most frequently in the parieto-occipital lobes [38]. Visual symptoms often resolve completely within hours or days; the resolution of neuroradiologic edema is longer than the clinical recovery period. In eclamptic patients, PRES is not the only possible explanation for seizures. Rarely, pregnant or postpartum women develop PRES for other reasons (such as drug use or RCVS) and not as a result of eclampsia. Thus, although there is overlap in most patients, eclampsia and PRES may occur independently [39].

Although the incidence of posterior reversible encephalopathy syndrome is high in eclamptic patients, nearly 20% of pre-eclamptic patients with neurological symptoms also develop posterior reversible encephalopathy syndrome [40]. Of eclampsia patients, 16% develop PRES, which is on the lower side of the range in the reviewed literature (10–90%). Eclampsia on presentation, recurrent seizures, postpartum eclampsia, cesarean delivery and labetalol use are associated with an increased risk of PRES development [41].

The therapeutic choice aims to provide good blood pressure control in such a way as to avoid the development of seizures or, in any case, to have the tools to manage them promptly. When we suspect pre-eclampsia, PRES or eclampsia, it is advisable to immediately start therapy with MgSO4, which resolves both hypertension and seizures [42]. Certainly, however, it is also necessary to undertake a typically antihypertensive treatment, the goal of which is to obtain a gradual return of blood pressure to the values of 140–155 mmHg for systolic and 90–105 mmHg for diastolic. These are the pressure ranges precisely because higher levels are associated with incomplete resolution of the edema. As antihypertensives, calcium-channel blockers administered parenterally and labetalol or nifedipine administered orally are mainly used [43]. Vasodilators are not used because they cause a worsening of the clinical picture that we observe in PRES [44]. Mannitol, an anti-edema agent, can also be used with the aim of reducing intracranial pressure [45]. 

### 2.4. Eclampsia

Eclampsia is defined as the occurrence of one or more generalized tonic–clonic convulsions unrelated to other medical conditions in women with hypertensive disorders of pregnancy [10]. It occurs in 0.8% of women with hypertensive disorders [46]. About 90% of eclampsia cases occur at or after 28 weeks of gestation. Just over one-third of eclamptic seizures occur at term and may develop intrapartum or within 48 h of delivery. So-called late or postpartum eclampsia, that is, eclampsia that begins more than 48 h after delivery, is increasingly reported. In the postpartum period, to date, from 11 to 55% of cases of pre-eclampsia and eclampsia have been diagnosed; this percentage could certainly increase if it were possible to recognize the picture in the pre-partum period. After giving birth, women tend to underestimate manifestations of possible pathologies, such as headaches and abdominal pain, and ask for medical advice only when the picture evolves towards critical situations, such as the presence of convulsive crises.

There are many factors associated with an increased risk of developing eclampsia, including Black and Hispanic race, an older mother, nulliparity, a mother aged ≤20, multifetal pregnancy, preterm delivery <32 weeks and a lack of adequate prenatal care [47].

Eclamptic crises are the hallmark of eclampsia and usually present as generalized tonic–clonic seizures lasting approximately 60 s. Convulsions may be preceded by clinical manifestations such as persistent frontal or occipital headache, blurred vision, photophobia in the right-upper quadrants, epigastric pain and altered consciousness. In approximately a third of the cases, hypertension and proteinuria are not reported before the crisis. There are several hypotheses regarding the mechanism of eclamptic seizures: Impaired cerebral vascular autoregulation in response to arterial hypertension could lead to arterial vasospasm and subsequent ischemia with cytotoxic edema. Alternatively, loss of autoregulation in response to arterial hypertension could lead to endothelial dysfunction, increasing capillary permeability with vasogenic edema. This vasculopathy may also cause PRES or regions of cerebral ischemia and hemorrhage. Although focal vasogenic edema is characteristic of eclampsia, up to a quarter of patients have areas of persistent cytotoxic edema corresponding to focal ischemia or hemorrhage. Therefore, areas of ischemia or hemorrhage in PRES and RCVS may contribute to eclamptic seizures.

The diagnosis of eclampsia is secure in the presence of generalized edema, hypertension, proteinuria and convulsions. However, women in whom eclampsia develops exhibit a wide spectrum of signs, ranging from severe hypertension, severe proteinuria and generalized edema to absent or minimal hypertension, no proteinuria and no edema. Hypertension is considered the hallmark of the diagnosis of eclampsia. This hypertension can be severe (at least 160 mm Hg systolic and/or at least 110 mm Hg diastolic) in 20–54% of cases or mild (systolic blood pressure between 140 and 160 mm Hg or diastolic blood pressure between 90 and 110 mm Hg) in 30–60% of cases. However, in 16% of the cases, hypertension may be absent. [48].

Although most women with typical eclampsia do not need neuroradiological investigations of the brain, postpartum eclamptic patients, particularly those with focal neurological deficits, persistent visual disturbances and symptoms refractory to magnesium and antihypertensive treatment, should undergo a comprehensive diagnostic evaluation, preferably including MRI [49]. Neuroimaging may also reveal areas of vasoconstriction consistent with RCVS. In rare cases, pregnant patients, especially those with RCVS, may develop craniocervical arterial dissection. Neuroimaging findings in patients with pre-eclampsia and eclampsia include infarction, hemorrhage, vasoconstriction, dissection and both vasogenic and cytotoxic edema [50].

When a patient is in critical condition, before being able to carry out a transfer, it is important to secure the patient, stabilize the arterial pressure and, obviously, work on the seizure. Therefore, it is necessary to administer a loading dose of magnesium and carry out fetal monitoring. It is advisable, during or after the convulsive episode, to provide for the maternal well-being, limiting any serious injuries. This is performed through the evaluation of the patient’s oxygenation status and making sure that the airways are clear, avoiding aspiration. Although the crisis lasts only a few minutes, it is important to administer supplemental oxygen through the use of devices such as a face mask at 8–10 L/min. If the condition of the pregnant woman becomes stable, it is possible to avoid an emergency cesarean delivery [10]. For preventing subsequent seizures in women with eclampsia, the drug of first choice is magnesium sulfate [51]. The recommended dosage is a loading dose of 6 g in 15–20 min and maintenance with 2 g every hour as a continuous IV solution. Maintenance with 2 g per hour aims to maintain magnesium at a therapeutic concentration of 543 with fewer fluctuations than with 1 g per hour [51]. Approximately 10% of women with eclampsia may develop a second seizure after receiving magnesium sulfate. In this case, it is recommended that a second bolus of 2 g of magnesium sulfate be given IV over 3 to 5 min. However, magnesium sulfate also has negative effects, caused by its ability to relax smooth muscles, which can lead to both respiratory depression and cardiac arrest. Deep tendon reflexes are lost at a serum magnesium level of 9 mg/dL (7 mEq/L), respiratory depression occurs at 12 mg/dL (10 mEq/L) and cardiac arrest occurs at 30 mg/dL (25 mEq/L). If there is an imminent risk of respiratory depression, intubate and carry out an emergency correction with a 10% calcium gluconate solution (10 mL IV over 3 min). Fishel Bartal (2020) recommends the need to monitor magnesium levels every 4–6 h only in women with renal dysfunction, i.e., with creatinine > 1.2 mg/dL or urine output that has been <30 mL/h for more than 4 h, as well as in women with signs of magnesium toxicity. If the serum level exceeds 9.6 mg/dL (8 mEq/L), the infusion should be stopped and serum magnesium levels should be determined at 2-hour intervals [10].

### 2.5. HELLP Syndrome

HELLP syndrome is a particular form of pre-eclampsia, described in 1982 by Weinstein as a nosological entity whose acronym is derived from the symptomatological triad “Hemolysis, Elevated Liver enzymes and Low Platelet count” [52].

The HELLP syndrome typically develops in the third trimester of pregnancy and/or in the first postpartum weeks, however, in 11% of the patients, the syndrome starts to manifest in the 27th week of gestation [53]. It is a clinical picture that can often present in association with pre-eclampsia, with an incidence ranging from 0.2 to 0.6% in pregnant women without hypertension [54]. In comparison, eclampsia has a reported incidence of 1.6 to 10 per 10,000 deliveries in developed countries, while it is 50 to 151 per 10,000 deliveries in developing countries [10]. HELLP occurs in 20% of patients presenting with pre-eclampsia or eclampsia [55]. It can also occur postpartum, with the onset in this case occurring within 48 h up to 7 days after delivery, even after a pregnancy that has not presented any apparent complications.

The variety of onsets can make diagnosis difficult. About 90% of patients have general malaise, 65% have epigastric pain, 30% have nausea and vomiting and 31% have headaches. Other onsets are scotomas, dyspnea, jaundice, hypertension and proteinuria [56].

The clinical presentation varies, and the only objective means of diagnosis and evaluation of the progression of the condition are laboratory tests. Even though the HELLP syndrome is considered a hypertensive multi-organ disorder of pregnancy, the level of hypertension does not correlate to the severity of the condition; hence, the diagnosis should be based on biochemical laboratory evidence. While arterial blood pressure is one of the main criteria in the diagnosis and treatment of pre-eclampsia, it is not as informative in the case of the HELLP syndrome and should not be used to predict its progression [57].

While there is not a current treatment for HELLP, the mainstay of treatment involves maternal stabilization and timely delivery. The use of corticosteroids has been studied, and Mao et al. (2015), through a meta-analysis, found that corticosteroid administration to HELLP patients improves platelet count and serum levels of LDH and ALT. It also reduces hospital/ICU stays and lowers blood transfusion rates. However, it is not significantly associated with improved maternal mortality and overall morbidity [58]. In 2020, Lokki et al. described a successful case of postpartum HELLP syndrome treated with the complement C5 inhibitor Eculizumab (900 mg IV) after the failure to respond to the best supportive care (plasma exchange treatment on the first and second postpartum days, as well as hemodialysis three times over the course of the treatment. Hypertension was treated with Amlodipine 10 mg twice a day and Labetalol 200 mg three times a day) [59].

### 2.6. Cerebral Cavernous Malformations (CCMs)

CCMs are vascular malformations of the brain, also known as cavernomas or cavernous angiomas, whose wall is composed of endothelial cells in the absence of connective tissue or muscle [60]. They represent 10–15% of all vascular malformations in the CNS [61], and they are mostly diagnosed between 30 and 39 years of age [62].

Cavernomas can be diagnosed as isolated lesions with a negative family history or as familial forms [63]. Familial cavernomatosis is associated with heterozygous loss-of-function (LOF) mutations in genes CCM1 (KRIT1), CCM2 or CCM3 (PDCD10) [64], and it is typically characterized by multiple lesions and a higher risk of bleeding compared to the sporadic form [65]. Susceptibility to CCM lesions is also related to inflammatory signals [66] and a permissive microbiome [67].

A diagnosis can be made accidentally through MRI performed for other reasons. The most common clinical manifestations include seizures (50%), intracranial hemorrhage (25%) and focal neurological deficits (FND) (25%) [68]. CCM hemorrhage is usually characterized by an acute onset of symptoms such as headache, epileptic seizure, impaired consciousness or new/worsening FND referable to the anatomic location of the CCM [69], although it was once believed that the possibility of bleeding increased during pregnancy [70] because of endothelial alterations caused by high levels of estrogen [71], leading to thrombosis and edema. To date, the evidence shows no statistical difference in the rates of hemorrhage between pregnant and non-pregnant patients [72].

MRI is the most commonly used method for the diagnosis of cavernomas, and it can be helpful to assess the risk of bleeding as well. In pregnancy, the correct evaluation is more difficult since gadolinium cannot be used [73].

Since seizures are the most common manifestation of CCM, antiepileptic therapy could be required. In pregnancy, maternal seizures can be dangerous for the fetus [74], and adequate counseling about the importance of reducing seizure worsening, setting up a medical therapy and assessing any teratogenic effect on the fetus must be performed [75]. Vaginal delivery is not contraindicated, even for patients who experience hemorrhagic stroke [76]. A multidisciplinary approach (gynecologist, anesthesiologist, neurologist, neurosurgeon, neuroradiologist) is needed in order to guarantee the lowest risk for the patient and to determine the actual need for neurosurgical procedures before delivery [77].

### 2.7. Stroke

Stroke is defined as an acute ischemic or hemorrhagic cerebrovascular event, and the incidence rate in the USA is approximately 30 per 100.000 during pregnancy and 14.7 per 100.000 during puerperium, which is 3 times higher than in non-pregnant women [78]. These numbers include strokes occurring after CVST, RCVS, amniotic fluid embolism, HELLP-related coagulopathy and all of the clinical pictures described in this study.

The first risk factor for stroke is older age; in fact, its incidence rate increases with increasing age [79]. Smoking is highly related to cerebrovascular disease and stroke [80]. Other medical conditions that are strongly associated with stroke include migraine, thrombophilia, LES, heart disease, sickle-cell anemia, hypertension (including gestational hypertension, pre-eclampsia and eclampsia [81]), thrombocytopenia and diabetes [82]. A recent cohort study showed an increased risk of ischemic stroke in women undergoing assisted reproductive techniques [83].

The most common physical findings in ischemic stroke are nausea, vomiting, paresthesia or weakness of arm or leg, facial weakness, speech disturbance, ataxic gait, headache, dizziness, paresis and abnormal eye movement [84].

Regarding diagnostic imaging, non-contrast study techniques, such as computed tomography (CT) and magnetic resonance imaging (MRI), are not contraindicated during pregnancy. Even though CT is based on the use of ionizing radiation, it allows us to make a rapid diagnosis, and this benefit overcomes any theoretical fetal risk, making it the test of first choice in emergencies with suspected acute stroke. The estimated fetal radiation exposure for a single maternal head CT is between 0.001 and 0.01 mGy, well below the safety threshold for the hypothetical development of teratogenicity or developmental disabilities. Conversely, magnetic resonance imaging is considered safe during pregnancy, but the limitation of this technique is that gadolinium is a class C drug, so its use is not recommended during gestation [85].

Maternal stroke is a medical and obstetrical emergency, so neurologists, emergency physicians, obstetricians, obstetrician anesthetists and interventional neuroradiologists and/or neurosurgeons can all be involved in the rapid assessment and decision-making required in this situation [86]. The first-line treatment for non-pregnant women with acute ischemic stroke is intravenous thrombolytic therapy and endovascular mechanical thrombectomy, but in pregnancy, these therapies have not been studied in randomized trials. The use of tissue plasminogen activator in pregnancy is recommended by the American Heart Association/American Stroke Association when the perceived benefits overweigh the risks [87]. An acute ischemic stroke associated with large-vessel occlusion is a severe risk for fetal and maternal health. The Canadian guidelines recommend endovascular thrombectomy, as the benefits almost always outweigh the risks [88].

The management of hemorrhagic stroke in pregnancy is similar to that of a non-pregnant patient. Coagulopathies should be corrected and blood pressure controlled [88].

The timing of delivery for women who have suffered a stroke during pregnancy is determined by the mother’s clinical condition and the stability of the fetus. It can be reasonable to begin the prophylaxis of the antenatal corticosteroid between 24 and 32 weeks of gestation, aiming for a controlled induction of labor between 34 and 39 weeks. A cesarean section should be avoided [89]. Secondary prevention of ischemic stroke in pregnancy and puerperium can be considered. Aspirin is the most widely used antithrombotic therapy.

### 2.8. Amniotic Fluid Embolism

Amniotic fluid embolism is a clinical condition that presents with agitation, confusion, seizures, encephalopathy and cardiovascular and respiratory insufficiency during or immediately after delivery. The scientific understanding of this condition, its pathophysiology and its management have all been historically hampered by a lack of uniform diagnostic criteria. In 2016, Clark et al. summarized the diagnostic criteria written by a committee of experienced specialists, such as representatives from maternal–fetal medicine, pulmonary/critical care medicine, hematology and obstetric anesthesiology [90]:

1. Sudden onset of cardiorespiratory arrest, or both hypotension (systolic blood pressure <90 mmHg) AND respiratory compromise (dyspnea, cyanosis or SpO2 < 90%). Women with AFE will typically experience almost simultaneous hemodynamic collapse and respiratory compromise reflecting primary cardiovascular and pulmonary insults, as well as an additional compromise of oxygenation secondary to the initial cardiovascular insult [90].

2. Documentation of overt disseminated intravascular coagulation (DIC) using the scoring system of the Scientific and Standardization Committee on Disseminated Intravascular Coagulation of the International Society on Thrombosis and Hemostasis (ISTH), modified for pregnancy [91]. Coagulopathy must be detected prior to the loss of sufficient blood to account for dilutional or shock-related consumptive coagulopathy. The presence of significant coagulopathy is one of the hallmarks that typically distinguish AFE from conditions such as myocardial infarction, anaphylaxis or drug reactions, anesthetic accidents and pulmonary thromboembolism.

3. Clinical onset occurs during labor or within 30 min of delivery of the placenta. This requirement is consistent with any condition involving acute inflammatory mediator release and would apply to typical as well as most atypical cases. Most cases will occur well before the 30 min window has elapsed, but clinical recognition may be delayed, especially during cesarean delivery under general anesthesia, due to ongoing routine respiratory and hemodynamic support [90].

4. No fever (T ≥ 38.0 °C) during labor is required in order to differentiate AFE from SIRS/SEPSIS [90].

In 2021, Simard et al. highlighted the role of echocardiography, in particular point-of-care ultrasound (POCUS), which has been shown to decrease the time to diagnosis and alter management for patients with dyspnea, shock or cardiac arrest [92].

### 2.9. Pituitary Apoplexy

Pituitary apoplexy, acute infarction and pituitary gland hemorrhage present with a clinical picture that is characterized by decreased consciousness and alertness, headache, ophthalmoplegia and even visual changes. They can also often occur in the context of an undiagnosed adenoma.

While it is accurate to say that marginal pituitary gland enlargement occurs during pregnancy, very rarely does pregnancy cause pituitary apoplexy. This pathology is divided into:

- Sheehan syndrome (pituitary ischemic necrosis): pituitary insufficiency (hypopituitarism) with onset after weeks/months of severe postpartum hemorrhage;

- Pituitary lymphocytic apoplexy: patients present with headache and visual symptoms; these are symptoms that also arise acutely in pregnant women and with a slow onset in non-pregnant women.

A brain MRI is certainly useful for making the diagnosis.

As for initial treatment, fluid and electrolyte replacement along with prompt replacement of deficient hormones and careful observation can be employed.

The latest guidelines [93] recommend hydrocortisone (100–200 mg) as an intravenous bolus, followed by a continuous intravenous infusion (2–4 mg per hour) or intramuscular injection every 6 hours (50–100 mg).

There are no clear recommendations regarding the treatment of pituitary apoplexy in pregnant women, especially regarding the choice between a conservative approach with dopamine agonists and neurosurgical intervention via transsphenoidal resection. Bromocriptine is the dopamine agonist of choice in the setting of pregnancy because of its greater safety record. Some authors [94] suggest that dopamine agonist therapy, even in the setting of a low prolactin level, may help prevent the normal increase in pituitary gland size during pregnancy.

Transsphenoidal surgery under general anesthesia does not appear to present a teratogenic risk to the fetus. Moreover, according to previous reports and our own experience, 19 cases underwent urgent decompressive pituitary surgery and had good outcomes in terms of symptoms and deliveries, suggesting that pituitary surgery for pregnant women is a feasible option. Most studies indicate that surgical treatment, usually within 7 days of the apoplectic event, leads to higher rates of visual impairment recovery [95].

## 3. Relevant Section

On the basis of what has been said about the different neurological conditions responsible for complications in pregnant women and the puerperium, some useful indications will now be provided in their diagnosis, starting from the common symptoms observable in the patients. Therefore, in this section, we want to provide a rapid diagnostic algorithm that is useful to the doctor in an emergency situation observable in the delivery room and that is therefore a support tool aimed at facilitating the management of these clinical situations.

### 3.1. Headache

In this brief discussion of the most represented acute neurological syndromes in pregnancy and puerperium, we have shown that headache is the most frequent clinical feature. A physician who has to manage patients who experience headaches during pregnancy or postpartum has to make a differential diagnosis among multiple medical conditions. Transient motor, sensory and visual deficits or speech disturbances in pregnant women are more likely to be attributable to hemicrania with aura, even in the absence of headache. In this case, neurological symptoms begin gradually and more often include positive phenomena (e.g., photopsies or scintillating scotomas) than negative ones (e.g., negative scotomas or, more rarely, partial/total visual field loss). Typically, symptoms resolve in one modality (e.g., visual) and then develop into another modality (e.g., somatosensory or aphasic). On average, each symptom regresses in 20 to 30 min.

The International Classification of Headache Disorders (ICHD) divides headaches into primary and secondary categories [96]. Primary headaches, including migraine and tension-type headaches, do not have a clear underlying causative pathology and are diagnosed according to their phenomenology. Secondary headaches are caused by an identifiable abnormality, and the list of etiologies for them is extensive. A large majority of the secondary headaches seen in pregnancy and the puerperium are caused by vascular disorders, in particular conditions associated with gestational hypertension [97].

The epidemiology of headaches during pregnancy and the postpartum period is very distinct from that in males or non-pregnant females, in part due to the hemodynamic and hematologic changes that occur during pregnancy. These changes put women at higher risk for cerebral venous thrombosis, hemorrhagic stroke and hypertensive disorders of pregnancy such as pre-eclampsia, eclampsia, posterior reversible encephalopathy syndrome and reversible cerebral vasoconstriction syndrome [98]. Headache in pregnancy and the puerperium can be a cause of heightened anxiety in most women, who are concerned not only about the effect of the headache itself on the pregnancy but also about the effect of the treatment options on the pregnancy and the fetus.

In the case of headaches during pregnancy or puerperium, the medical approach should start with an accurate anamnesis. It is important to ask the patient if she is experiencing a sudden-onset severe headache (thunderclap headache) or if the headache is unusual and there is a change in the pattern. Furthermore, in the patient’s personal and family medical history, previous cerebrovascular diseases should be carefully investigated. Other signs and symptoms that should be investigated are nausea, vomiting, visual disorders, hypertension (blood pressure > 140/90 mmHg), papilledema, confusional state, fever and focal neurological signs (paresthesia or weakness of arm or leg, facial weakness, speech disturbance, ataxic gait, paresis, abnormal eye movement, etc.) [99].

Thunderclap headache (TCH) is an abrupt onset of severe headache that needs to be thoroughly investigated because the most common secondary cause is subarachnoid hemorrhage (SAH) [99]. The first step to investigating an SAH is to perform a cerebral CT without contrast medium.

**Figure 1:**
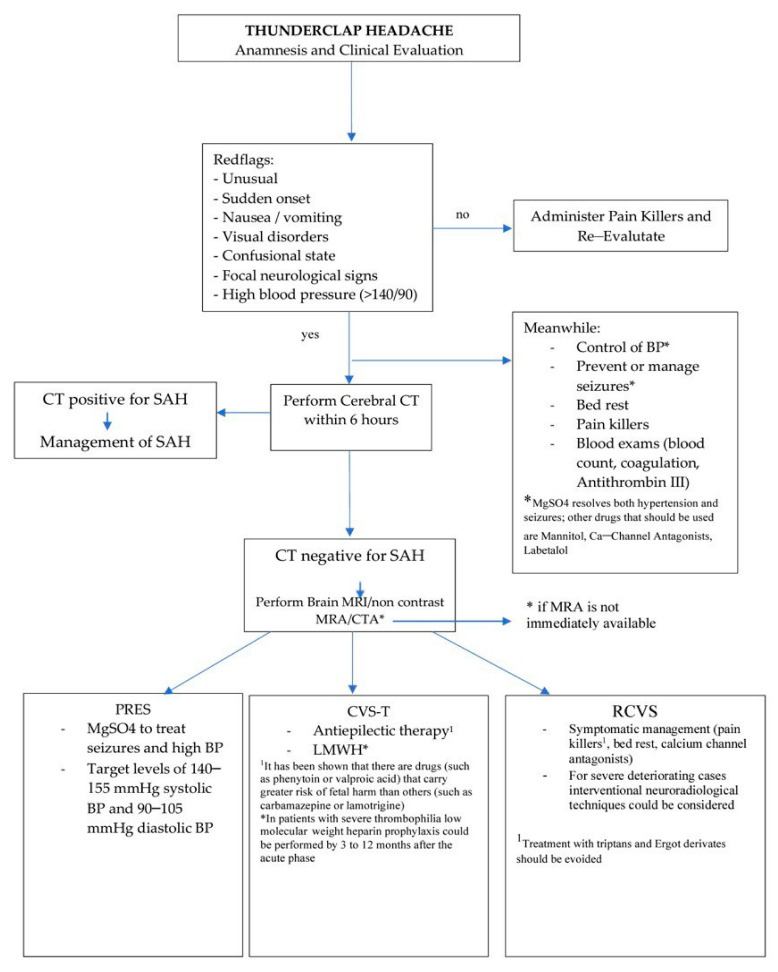
Algorithm for managing thunderclap headache in a clinical setting.

The dosage of a single CT scan is compatible with pregnancy. No evidence of increased fetal risk has been reported for an exposure of less than 100 mGy, and the fetal exposure of an abdomen CT scan is approximately 10–50 mGy and that of a head and cervical spine CT scan is approximately 1–10 mGy [100]. The theoretical risk of fetal carcinogenic induction possibly causing juvenile cancer when exposed to ionizing radiation from a single CT examination is estimated to be 1/1000 to 1/500 (from 0.1 to 0.2%); however, the only study on this subject in the literature did not report a significantly increased risk of fetal carcinogenesis in women exposed to ionizing radiation compared with women not exposed to ionizing radiation from CT during pregnancy [101]. If the CT is negative for SAH, other clinical conditions have to be excluded, such as PRES, CVT and RCVS. These diseases, as we discussed in the previous sections, may not always be visible with a CT, so one should proceed with another imaging technique such as an encephalic MRI. In the case of PRES, MRI shows focal edema, most frequently in the parieto-occipital lobes. In the case of CVT, a non-contrast brain MRI or MRV is the first-choice diagnostic investigation that shows signs of infarction that often undergo hemorrhagic transformation. In the case of RCVS, if CT and MRI do not show any sign, a segmental cerebral artery vasoconstriction can be seen on a Magnetic Resonance Angiography (MRA); the gold standard for its diagnosis is Digital Subtraction Angiography (DSA), but it is an invasive imaging technique and not always available. In Figure 1, a brief useful algorithm to follow in the above-mentioned clinical situation is presented.

### 3.2. Seizures

Status epilepticus (SE) is a single epileptic seizure lasting more than five minutes or two or more seizures occurring within a five-minute period without the person returning to normal between them [102].

SE has been reported in 0.6% of all pregnancies, and convulsive status in 0.3% [103].

Epilepsy in pregnancy carries significant risk to both the mother and the fetus, and it is associated with high mortality and morbidity. It is very important that the treatment of epilepsy not be delayed because, in addition to the risk imposed on the mother, prolonged SE can compromise placental blood flow and cause fetal hypoxia [74].

Eclampsia is presumed to be the most common overall cause of generalized tonic–clonic seizures occurring in gestation, labor or puerperium [104]. In addition to eclampsia, other conditions such as viral encephalitis, systemic lupus erythematosus, cavernoma, reversible cerebral vasoconstriction syndrome and pyridoxine deficiency have been associated with SE in pregnancy [105].

**Figure 2:**
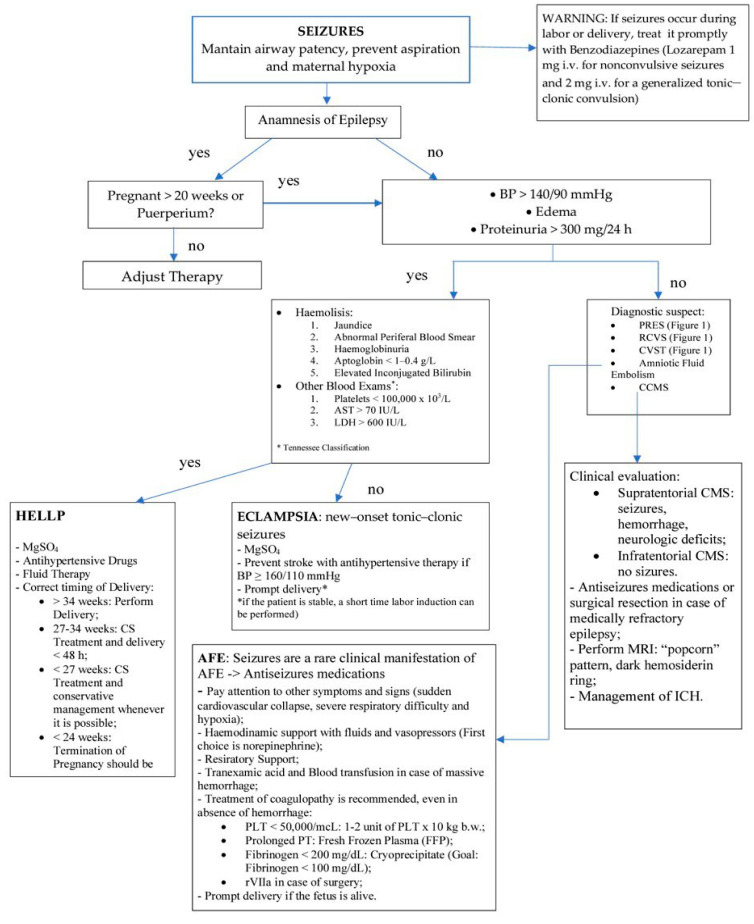
Algorithm for managing seizures in the clinical setting.

All pregnant or postpartum women presenting with a new-onset seizure disorder are expected to undergo specific diagnostic investigations, including a brain MRI, as brain CT has reduced sensitivity. On the other hand, when the convulsive crisis is attributable to a picture of prenatal eclampsia, it is possible to bypass the execution of neuroimaging and follow the specific treatment. Figure 2 presents a concise and useful algorithm to follow in the clinical situation mentioned above.

### 3.3. Acute Neurological Deficit

When a pregnant woman or a woman in the puerperium presents symptoms of the motor, visual or sensory component, further tests should be performed, especially when these symptoms are acute and tend to persist. It is necessary to investigate to exclude serious pathologies. In cases where the visual component is prevalent, the pathologies that may affect it are PRES, pituitary apoplexy and stroke. It is also possible for orbital hemorrhages to manifest themselves with diplopia, proptosis and ocular pain, typically acute; the latter is a typical situation in the first trimester due to hyperemesis or during delivery due to pushing.

Stroke in pregnant and postpartum women is rare, but the risk is higher than in non-pregnant women of the same age, especially in late pregnancy and early puerperium. [76] The period in which women are at greatest risk of developing stroke is from the third trimester up to 6 weeks postpartum [106]. Pre-eclampsia and eclampsia are the cause in 25–50% of ischemic stroke cases [81]. Other risk factors are age, African-American race, hypertension, heart disease, cesarean delivery, migraine, thrombophilia, thrombocytopenia, sickle-cell disease and systemic lupus erythematosus. The most common physical findings in ischemic stroke are nausea, vomiting, paresthesia or weakness of arms or legs, facial weakness, speech disturbance, ataxic gait, headache, dizziness, paresis and abnormal eye movement [84].

Regarding diagnostic imaging, non-contrast tests, including computed tomography (CT) and magnetic resonance imaging (MRI), are not contraindicated during pregnancy.

Dissection of the cervicocranial arteries can also cause strokes during pregnancy, in which case patients will have isolated nuchal headache without neurologic deficits; however, symptomatic cerebral infarctions may develop.

Additionally, performing the Valsalva maneuver during delivery or malpositioning the patient (especially if hyperextended neck during anesthesia occurs) may also be risk factors for stroke, although there is no evidence for this [107]. In patients with cerebral hemorrhage and subarachnoid hemorrhage, the presence of vascular malformations and aneurysms is relatively common. Subarachnoid hemorrhages that develop near the circle of Willis often imply that they are caused by underlying aneurysms, whereas subarachnoid hemorrhages of the convexity are more often caused by RCVS or CVT. Rare causes of stroke in postpartum pregnant women also include thrombotic thrombocytopenic purpura, pituitary apoplexy, amniotic embolism, choriocarcinoma, air embolism and cardioembolism from postpartum cardiomyopathy. The timing of delivery for women who have suffered a stroke during pregnancy is determined by the clinical condition of the mother and the stability of the fetus. It can be reasonable to begin the prophylaxis of the antenatal corticosteroid between 24 and 32 weeks of gestation, aiming for a controlled induction of labor between 34 and 39 weeks. Cesarean sections should be avoided [108].

**Figure 3:**
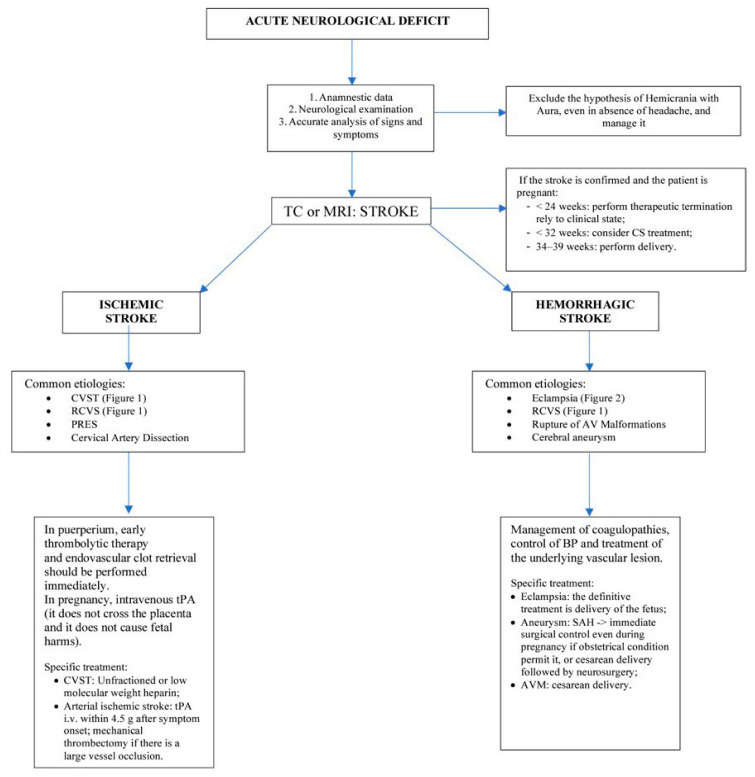
Algorithm for managing acute neurological deficit in the clinical setting.

There is a paucity of literature regarding the preferred mode of delivery for women with underlying neurosurgical and neurological conditions. In women with evidence of raised intracranial pressure due to intracranial space-occupying lesions with mass effect or obstruction of CSF flow at or above the foramen magnum, neuraxial anesthesia is associated with a significant risk of herniation, and delivery should be performed by CS using general anesthesia. The preferred mode of delivery for women with VHL needs to be based on the assessment of each individual’s clinical features by a multidisciplinary team. In other chronic neurosurgical and neurological disorders, the available evidence suggests the mode of delivery should be based upon obstetric indications. Large prospective studies assessing maternal and fetal outcomes are needed to establish guidelines regarding the preferred mode of delivery with neurosurgical and neurological conditions [108]. Figure 3 presents a brief and useful algorithm to follow in the above-mentioned clinical situation.

## 4. Conclusions and Future Directions

The management of cerebral emergencies in pregnant patients is based on a multidisciplinary approach involving several specialists (gynecologists, anesthesiologists, neurologists and neurosurgeons). An incorrect diagnosis and/or failure to identify the symptoms and risk factors associated with the main neurological alterations during pregnancy would lead to a delay in the correct therapeutic approach, increasing the danger of death or severe neurological damage in the mother and the fetus. Therefore, an adequate and in-depth knowledge of the main neurological pathologies, their respective risk factors and the physiological changes during pregnancy are fundamental elements to making a correct diagnosis and directing the safest therapeutic path for the patient and the unborn child.

## Data Availability

Data sharing not applicable.
